# MitosRNAs and extreme anoxia tolerance in embryos of the annual killifish *Austrofundulus limnaeus*

**DOI:** 10.1038/s41598-019-56231-2

**Published:** 2019-12-24

**Authors:** Claire L. Riggs, Steven Cody Woll, Jason E. Podrabsky

**Affiliations:** 1 Brigham & Women’s Hospital/Harvard Medical School, Rheumatology, Department of Medicine, The Hale Building for Transformative Medicine, 60 Fenwood Road, Boston, MA 02115 USA; 2 Oregon Health and Science University, School of Medicine, 3181 SW Sam Jackson Park Rd, Portland, OR 97239 USA; 3 Department of Biology, Portland State University, P.O. Box 751, Portland, OR 97207 USA

**Keywords:** Small RNAs, Mitochondria

## Abstract

Embryos of the annual killifish *Austrofundulus limnaeus* are the most anoxia-tolerant vertebrate. Annual killifish inhabit ephemeral ponds, producing drought and anoxia-tolerant embryos, which allows the species to persist generation after generation. Anoxia tolerance and physiology vary by developmental stage, creating a unique opportunity for comparative study within the species. A recent study of small ncRNA expression in *A*. *limnaeus* embryos in response to anoxia and aerobic recovery revealed small ncRNAs with expression patterns that suggest a role in supporting anoxia tolerance. MitosRNAs, small ncRNAs derived from the mitochondrial genome, emerged as an interesting group of these sequences. MitosRNAs derived from mitochondrial tRNAs were differentially expressed in developing embryos and isolated cells exhibiting extreme anoxia tolerance. In this study we focus on expression of mitosRNAs derived from tRNA-cysteine, and their subcellular and organismal localization in order to consider possible function. These tRNA-cys mitosRNAs appear enriched in the mitochondria, particularly near the nucleus, and also appear to be present in the cytoplasm. We provide evidence that mitosRNAs are generated in the mitochondria in response to anoxia, though the precise mechanism of biosynthesis remains unclear. MitosRNAs derived from tRNA-cys localize to numerous tissues, and increase in the anterior brain during anoxia. We hypothesize that these RNAs may play a role in regulating gene expression that supports extreme anoxia tolerance.

## Introduction

Embryos of the annual killifish *Austrofundulus limnaeus* are the most anoxia-tolerant vertebrate known^1^. The most tolerant embryonic stages survive over 100 days without oxygen^1,2^. During embryonic development, embryos range from anoxia-sensitive to highly anoxia-tolerant, allowing an opportunity for comparative study of phenotypes within the species^1^. Metabolic depression is central to surviving anoxia in *A*. *limnaeus*^3^, as well as in the few other anoxia-tolerant vertebrates^4^. Therefore, understanding the cellular mechanisms that support entry into and exit out of metabolic depression is of particular interest. A recent revolution in small non-coding RNA (small ncRNA) research has demonstrated that small ncRNAs play pivotal roles in practically every aspect of cell and organismal physiology, particularly by regulating gene expression^5,6^. A study on small ncRNAs in embryos of *A*. *limnaeus* revealed unique expression patterns associated with different anoxia-tolerance phenotypes (i.e. embryonic stages)^7^. While many miRNAs, the most well-studied class of small ncRNAs, were differentially expressed in response to anoxia and recovery, a very interesting expression signature was identified for mitosRNAs, a class of small ncRNAs derived from the mitochondrial genome^7^. In actively developing embryos that exhibit extreme anoxia tolerance, anoxia strongly increased the abundance of mitosRNAs. In contrast to the other classes of small ncRNAs, many mitosRNAs reach their highest abundance during anoxia and not during recovery. As embryos developed past this stage and start to lose their anoxia tolerance the mitosRNA response was muted. The distinct expression pattern of mitosRNAs within *A*. *limnaeus* strongly suggests that mitosRNAs may be critical to supporting extreme anoxia tolerance in embryos of *A*. *limnaeus*^7^. Here we further investigate the nature of mitosRNAs in *A*. *limnaeus*, including probing their subcellular and whole embryo localization, as well as possible mechanisms for their biosynthesis.

Mitochondria-derived small non-coding RNAs were first identified in isolated mouse mitochondria^8^ and in the mitochondria of a flagellated protozoan parasite^9^. A later study on small RNAs associated with human mitochondria identified “putative novel miRNAs”, aligning to regions of the mitochondrial genome, including 16S rRNA, tRNAs, and subunits of complex I^10^. This same study confirmed the association of miRNAs, piRNAs, and snoRNAs with mitochondria, yet their genomic origin was uncertain. The term mitosRNAs was coined by Ro *et al*.^11^ in 2013, in their study of small ncRNAs in mouse and human mitochondrial (mt) genomes, where they identified 1000s of small ncRNAs encoded by the mt genome. Ro *et al*. identified mitosRNAs derived from all regions of the mitochondrial genome: protein-coding, rRNA, tRNA, and non-coding, and confirmed subcellular localization to the mitochondria by northern blot assay. The function of mitosRNAs remains uncertain, though a detailed study of mitosRNA-1978 in CHO (chinese hamster ovary) cells has demonstrated a miRNA-like role, where the small ncRNA regulates gene expression of two genes localized to the endoplasmic reticulum^12^. Ro *et al*. found that mitosRNAs target antisense transcripts and enhance the expression of mitochondrial genes which they annotate to^11^.

Since the discovery of mitosRNAs, a few studies have identified differentially expressed mitosRNAs in non-traditional model organisms. In the tadpole shrimp *Triops cancriformis*, an analysis of tRNA-derived fragments (tRFs) identified tRFs derived from 16 different mitochondrial tRNAs^13^, whose expression varied over development^13^. MitosRNAs were also identified in eggs of the rainbow trout, with a high proportion originating from mitochondrial tRNAs^14^. Post-ovulatory aging of the eggs resulted in decreased expression of the mitosRNAs. MitosRNAs are also present in chicken breast muscle, and results suggest they may play a role in muscle growth^15^. While these studies indicate that differential expression of mitosRNAs may play a role in growth and development, the study on anoxia tolerant annual killifish was the first to document mitosRNAs, many of which were derived from tRNAs, changing in abundance in response to stress^7^. Here we focus on these mitosRNAs in annual killifish embryos, which vary in their anoxia-tolerance levels across development.

The range of anoxia-tolerance levels and phenotypes in *A*. *limnaeus* presents a unique opportunity for comparative study, allowing us to assess if mitosRNAs may be critical for surviving anoxia, and to explore the potentially adaptive roles of these novel sequences in this context. *A*. *limnaeus* embryos can enter metabolic depression associated with diapause at 3 distinct developmental stages termed diapause 1, 2, and 3^16,17^. Diapause 2 (D2) embryos are metabolically depressed and exhibit the maximum anoxia-tolerance displayed in embryos of *A*. *limnaeus*, surviving over 100 days without oxygen^2^. Note, post-D2 staging can be identified by the number of days post-diapause (dpd) or based on morphology as described by J.P. Wourms^16,18^. As embryos exit D2 and resume active development, extreme anoxia tolerance is retained for at least 4 days of post-diapause 2 (4 dpd) development until the embryos reach Wourms’ Stage (WS) 36. After WS 36, anoxia tolerance declines as the embryos develop towards hatching. By 12 dpd at WS 40, embryos survive about 12 days without oxygen and by 20 dpd (WS 42) embryos only survive about 24 hours of anoxia^1,2^. Additionally, the anoxia tolerance of WS 40 embryos can be extended by about 30% with a 24 h non-lethal period of anoxic preconditioning^1^. The comparison between D2 (WS 32/33) and WS 36 embryos provides a unique opportunity to compare dormant and metabolically active embryos that share the same remarkable anoxia tolerance. MitosRNA expression patterns in context of this biology suggest that they may be critical to supporting anoxia tolerance and therefore deserve further investigation^7^. In this study we examine the expression, localization, and possible mechanism of generation of mitosRNAs in anoxia-tolerant *A*. *limnaeus* embryos and in an anoxia-tolerant cell line derived from *A*. *limnaeus* embryos.

## Results

### mitosRNAs are differentially expressed over development and in response to anoxia

**Figure 1:**
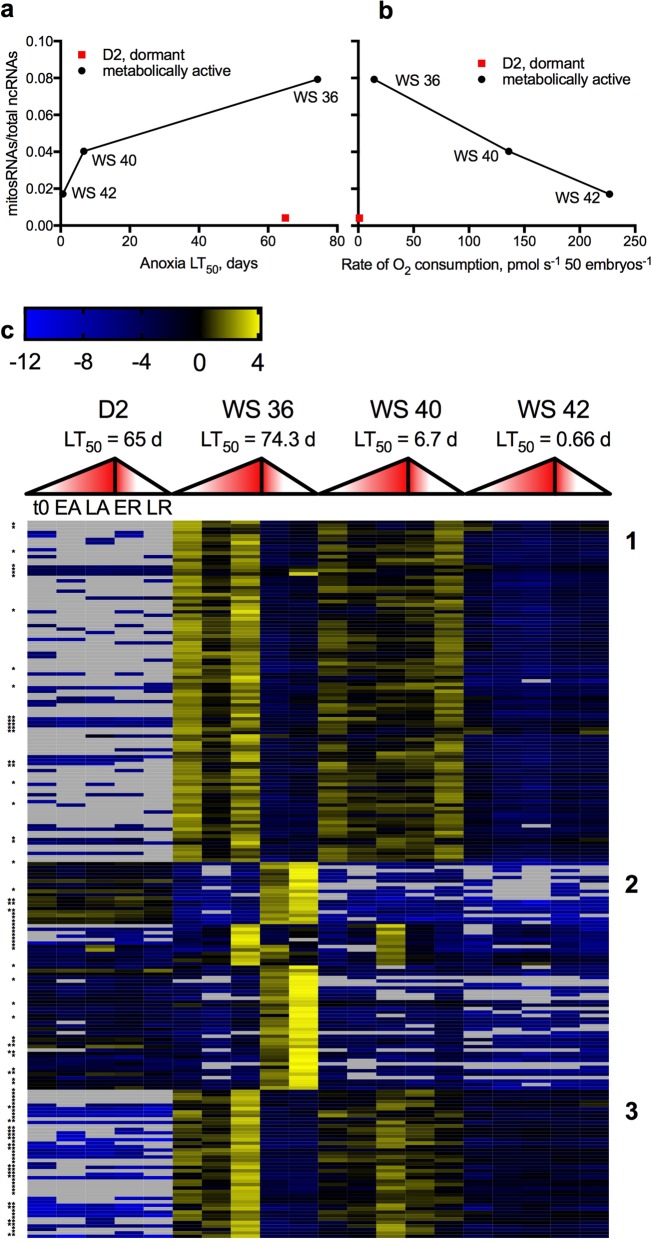
MitosRNA expression over development and in response to anoxia in *A*. *limnaeus* embryos reveals putative relationship between mitosRNA expression and anoxia tolerance. (**a**) Line graphs of the sum of mitosRNA expression relative to the sum of the expression of all small ncRNAs identified during normoxia within embryonic stages differing in anoxia LT_50_^2,7^. (**b**) Line graphs displaying relative mitosRNA expression (see a) with corresponding metabolic rate of each embryonic stage^17^. (**c**) Heatmap of all mitosRNAs differentially expressed (normalized mean expression across all samples >25, log_2_ fold change >2, adjusted p < 0.01) in response to anoxia and recovery in at least one embryonic stage studied. Expression patterns fall into 3 broad expression clusters. Log_2_ fold change values are calculated relative to the mean expression across all samples. Yellow indicates an increase in abundance, while blue indicates a decrease in expression relative to the mean. Missing values are shown in grey. Expression patterns are displayed for each stage for comparison, even though mitosRNAs may not be differentially expressed in all embryonic stages. Expression on the heatmap corresponds with the five treatments from left to right as indicated for D2 embryos (t0 = time zero; EA = early anoxia, 4 h of anoxia for D2, WS 36 and WS 40 embryos and 2 h for WS 42; LA = long anoxia, 24 h of anoxia for D2, WS 36, and WS 40 embryos and 6 hr for WS 42; ER = early recovery, 2 h of aerobic recovery for all stages; LR = late recovery, 24 h of recovery for all stages). The mid point of the gradient filled triangles marks the transition from LA to ER for each stage. Single asterisk (*) indicates mitosRNAs annotating as fragments of tRNAs and double asterisk (**) indicates mitosRNA annotating to tRNA-cys.

**Figure 2:**
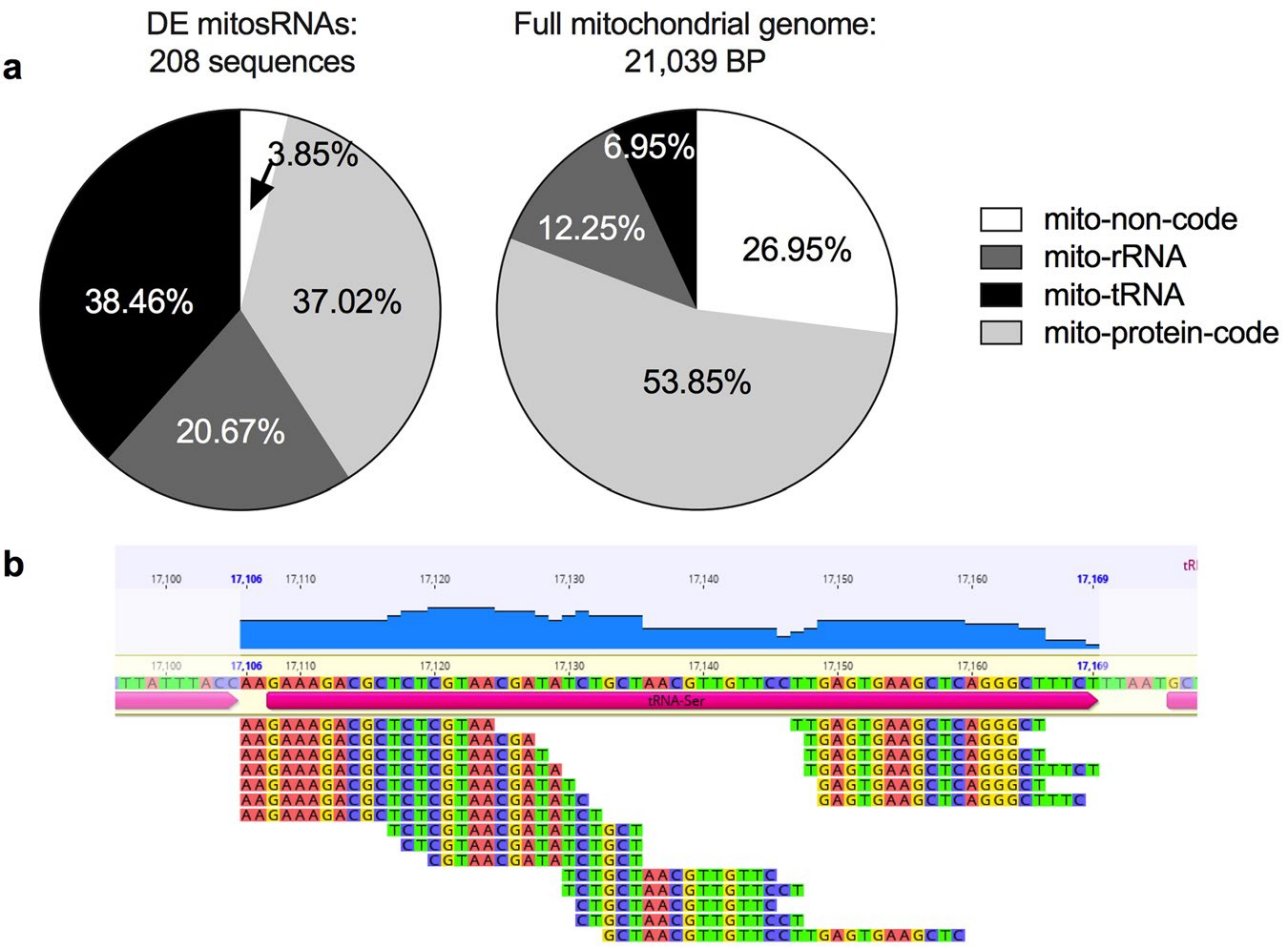
Differentially expressed mitosRNAs align to coding and non-coding regions of the mitochondrial genome and are enriched for tRNAs. (**a**) Annotation distribution of differentially expressed mitosRNAs that map to the mitochondrial genome and the percentage of the mitochondrial genome coding for each gene type. Differentially expressed mitosRNAs annotate throughout the mitochondrial genome non-coding, protein, tRNA, and rRNA-coding regions, and some extend into intergenic regions. (**b**) Screen shot from Geneious software showing small ncRNAs annotating to tRNA-ser upstream of the transcription start site for the gene. Data are from Riggs & Podrabsky 2017^7^.

Overall levels of mitosRNA expression are positively correlated with anoxia tolerance (Fig. 1a, r = 0.95, p = 0.19) and negatively correlated with metabolic rate (Fig. 1b, r = −0.99, p = 0.039) of metabolically active embryos. However, in dormant D2 embryos the proportion of mitosRNAs relative to total small ncRNAs is very low despite their high anoxia tolerance and low metabolic rate (Fig. 1a,b). Even when exposed to anoxia and recovery, D2 embryos still lack a robust mitosRNA response (Fig. 1c). Conversely, WS 36 embryos, the most anoxia-tolerant developing stage, show a pronounced increase in abundance of mitosRNAs when exposed to anoxia followed by aerobic recovery (Fig. 1c). There are 2 dominating patterns in WS 36 embryos, increased abundance during anoxia and increased abundance during recovery. WS 40 and WS 42 embryos share differential expression of some of the same mitosRNAs identified in WS 36 embryos, however, with lower changes in abundance (Fig. 1c). Differentially expressed mitosRNAs are derived from tRNA, rRNA, protein-coding, and non-coding regions of the mitochondrial genome (Fig. 2a, Table 1) and do not reflect proportions of the mitochondrial genome coding for each type of gene (Fig. 2a). Some mitosRNAs identified span multiple mitochondrial genes or extend into intergenic regions, such as mitosRNAs that annotate 2 nucleotides upstream of tRNA-ser and extend into the tRNA (Fig. 2b). MitosRNAs align to sequences on both the heavy and light strands of the mitochondrial genome, with the majority coming from the heavy strand (Table 1).

**Table 1:** Alignment information for mitosRNAs identified in *A*. *limnaeus* embryos.

	Alignment	Strand	Total mitosRNAs	tRNAs	rRNAs	mRNAs	D-loop	Origin of replication	Intergenic
Differentially expressed mitosRNAs	Single feature	Heavy	140 (67.3%)	33	42	65	NA	NA	0
		Light	52 (25.0%)	39	0	5	4	4	0
	Overlapping features	Heavy	15 (7.2%)	7	1	7	NA	NA	0
		Light	1 (0.5%)	1	0	0	0	0	0
mitosRNA catalog	Single feature	Heavy	75008 (85.0%)	2702	15065	57241	NA	NA	0
		Light	9031 (10.2%)	2349	0	2567	3975	140	0
	Overlapping features	Heavy	3484 (3.9%)	522	1423	571	872	18	78
		Light	761 (0.9%)	146	61	50	415	87	2

### Anoxia-responsive mitosRNAs are enriched for small ncRNAs annotating to mito-tRNAs

While mitosRNAs encoded in many different regions of the mitochondrial genome are differentially expressed in response to development and exposure to anoxia and recovery, mitosRNAs derived from tRNAs comprise a high proportion of those differentially expressed in response to anoxia and recovery (Figs. 1c and 2a, Table 1). Over 38% of mitosRNAs are derived from mitochondrial tRNAs (sequences marked with an asterisk in Fig. 1c), and many of the most highly differentially expressed sequences belong to this group. As has already been pointed out in a previous paper, there is an enrichment for tRNA-derived small ncRNAs (Fig. 2a) compared to their representation in the mitochondrial genome as well as the catalog of all *A*. *limnaeus* small ncRNA sequences identified^7^.

### MitosRNAs derived from mitochondrial tRNA-cys are highly anoxia-responsive

**Figure 3:**
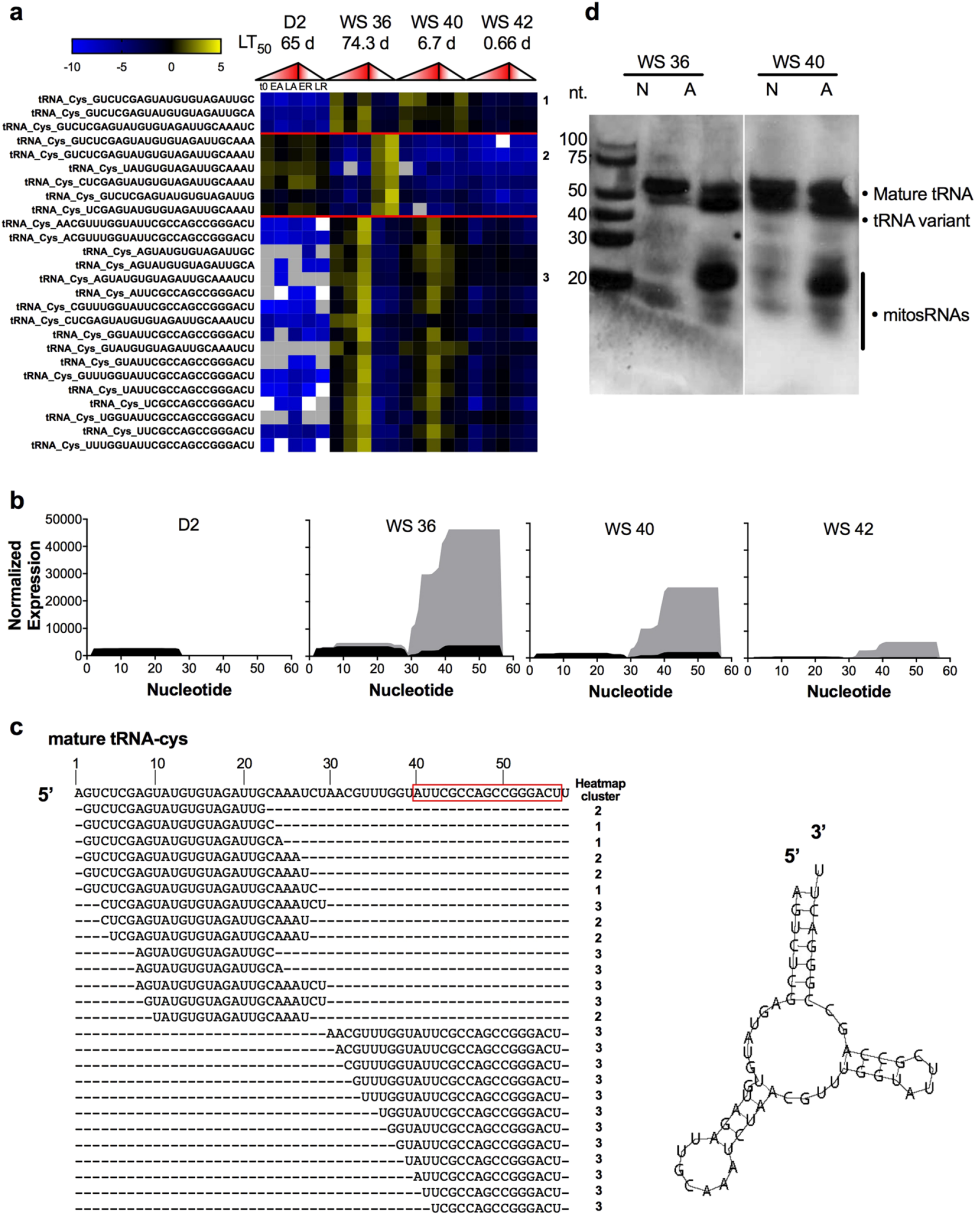
MitosRNA-tRNA-cys variants are strongly induced in response to anoxia in developing embryos with high tolerance of anoxia. (**a**) Heat map of differentially expressed mitosRNA-tRNA-cys sequences. Refer to Fig. 1 for heatmap details. There are 3 main clusters of expression for mitosRNA-tRNA-cys indicated on the right side of the heat map and described in the text. **(b**) Graphs illustrating the sum of normalized read counts (y axis) plotted for each base of the tRNA (x axis) at each stage of development. Black indicates read expression during normoxia and grey indicates read expression during late anoxia (24 h anoxia for D2, WS 36, and WS 40 embryos; 6 h anoxia for WS 42 embryos). **(c)** Mature tRNA-cys and alignment of differentially expressed mitosRNAs-tRNA-cys variants. The red box around a portion of the mature tRNA indicates the probe target. Stem-loop structure for mature mitochondrial tRNA-cysteine generated by MITO as previously published^21^ is shown next to the alignment. **(d)** Northern blot illustrating that mitosRNA-tRNA-cys RNAs are strongly enriched/upregulated during anoxia in WS 36 and WS 40 embryos. The band around 20 nucleotides represents variants of mitosRNA-tRNA-cys 3′ variants, displayed in the heat map. 3 µg RNA was loaded per well and the blot was hybridized with 0.5 nM mitosRNA-tRNA-cys probe directed to the 3′ end of the mature tRNA. Note that the two blots shown are from the same blot, but duplicate lanes run with a higher RNA load are not shown.

MitosRNAs derived from mitochondrial tRNA-cys that increase in expression in response to anoxia in WS 36 and WS 40 embryos (Figs. 1c and 3a) were chosen for follow-up work, due to their high abundance and large change in abundance in response to anoxia. The mitosRNAs derived from tRNA-cys cluster into 3 main patterns based on their expression. Cluster 1 is comprised of mitosRNAs that are abundant in WS 36 (4 dpd) embryos with a pattern of high abundance during normoxia, followed by a decrease in abundance after 4 h of anoxia, then a return to high expression after 24 h anoxia. Cluster 2 sequences are constitutively expressed in D2 embryos and increase in expression during recovery from anoxia in WS 36 embryos (Fig. 3a). Cluster 3, the largest cluster of mitosRNA-tRNA-cys sequences, increases in expression at 24 h of anoxia robustly in WS 36 embryos and in a more muted manner in WS 40 embryos (Fig. 3a). There is not a striking pattern correlating specific sequence variants with specific clusters. While all 3′ end-aligning mitoRNA-cys variants are found in cluster 3, some of the variants aligning to the 5′ end are also present in cluster 3. All three sequences found in cluster 1 only differ by the last several nucleotides, however other similar sequence variants are found in cluster 2, indicating that sequence expression patterns can vary widely for highly similar variants. Differentially expressed mitosRNA-tRNA-cys fragments are derived from both the 3′ and 5′ ends of the mature tRNA-cys, but the predominance of sequences in high abundance at 24 h anoxia align with the 3′ end of the mature tRNA (Fig. 3b,c). Therefore a probe was designed to capture the 3′ tRNA-cys mitosRNA variants (shown by the red box in Fig. 3c) for further analysis. Qualitative northern blot analysis using this probe confirms induction of mitosRNAs derived from the 3′ end of tRNA-cys in response to anoxia in WS 36 and WS 40 embryos (Fig. 3d), and revealed three distinct bands representing: (1) the mature tRNA, (2) a slightly faster migrating variant of the mature tRNA, and (3) the mitosRNA-tRNA-cys variants whose expression is induced by anoxia (Fig. 3d). The dominant band representing the small non-coding RNAs annotating to mito-tRNA-cys 3′ variants appears strongly under anoxia, a bit longer than 20 nucleotides long, which is consistent with deep sequencing data from cluster 3 of Fig. 3a.

### Anoxia induces expression of mitosRNAs, including mitosRNA-tRNA-cys in the PSU-AL-WS40NE cell line

**Figure 4:**
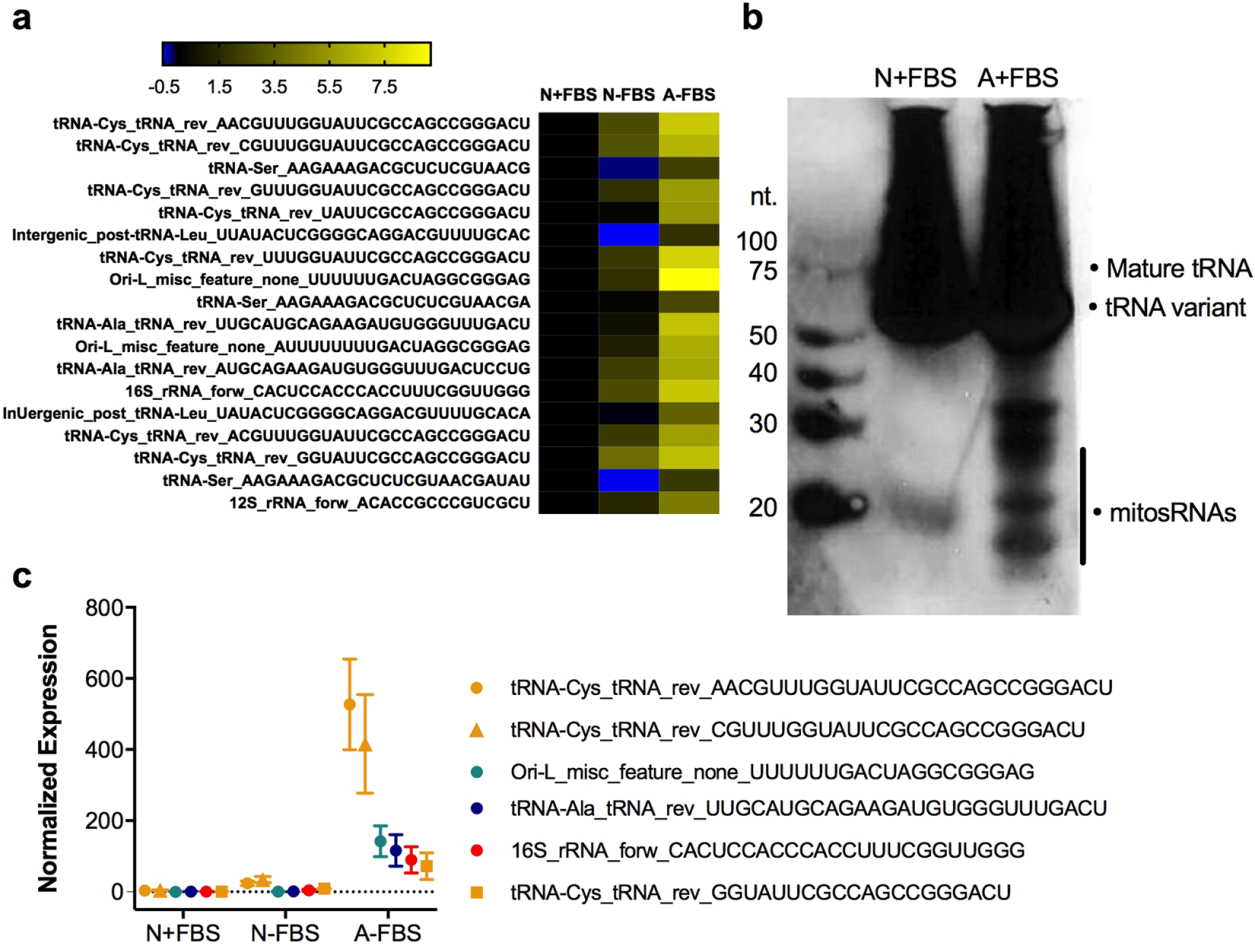
MitosRNA-tRNA-cys sequences are present and induced in response to anoxia in the *A*. *limnaeus* WS40NE cell line. (**a**) Heat map of mitosRNAs differentially expressed in response to anoxia in WS40NE cells. Log_2_ fold change is calculated relative to normoxic control with fetal bovine serum (N + FBS). For heat map details see Fig. 1 caption. (**b**) MitosRNAs-tRNA-cys 3′ variants northern blot of normoxic and 24 h anoxic WS40NE cells shows increased expression of a variety of mitosRNAs derived from tRNA-cys during anoxia. 20 µg RNA was loaded for normoxic and anoxic cells and the blot was hybridized with 2 nM of mitosRNA-tRNA-cys probe. Note: smear of RNA at top of blot is due to need to load high amount of RNA in order to get a signal for the mitosRNAs. This is likely due to relatively low expression of mitosRNAs in the cells. (**c**) Line graphs of the 6 most highly differentially expressed mitosRNAs in response to anoxia. Note appearance of 3 variants of mitosRNAs annotating to mitochondrial tRNA-cys.

The anoxia tolerant PSU-AL-WS40NE (henceforth referred to as WS40NE) cell line is derived from WS 40 *A*. *limnaeus* embryos and can survive at least 7 weeks of anoxia^19^. Small RNA sequencing revealed the presence and expression of mitosRNAs in this cell line, and while not all mitosRNAs identified in whole embryos are present in the WS40NE cells, a subset are highly induced in response to anoxia (Fig. 4a^19^). Like in whole embryos, many of these sequences annotate to mitochondrial tRNAs. MitosRNA-tRNA-cys sequence expression, like in whole embryos, is also highly induced in response to anoxia in the cell line (Fig. 4). Northern blot analysis of WS40NE cells reveals multiple size classes of mitosRNA-tRNA-cys sequences induced by anoxia in WS40NE cells (Fig. 4b,c). The band around 20 nucleotides corresponds with variants identified in the small RNA sequencing data (Fig. 4a–c).

### mitosRNAs-tRNA-cys subcellular localization

**Figure 5:**
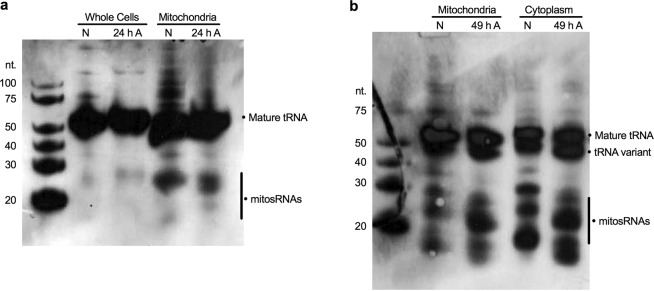
Northern blot of subcellular localization of mitosRNA-tRNA-cys 3′ variants in the WS40NE cell line. 5 µg RNA was loaded in each well and blots were hybridized with 0.5 nM mitosRNA-tRNA-cys probe. Cells were treated with anoxia for 24 or 49 h. (**a**) mitosRNAs derived from tRNA-cys, around 20–30 nucleotides long, are enriched in the mitochondrial fraction and in response to anoxia compared to whole cells. Note, mitosRNA band is not very strong in the northern blot of the whole cells here (4a) while they are clearly present in 3b. This discrepancy is due to the difference in loading 5 µg RNA for Fig. 5a vs 20 µg RNA for Fig. 4b. (**b)** Subcellular fractionation suggests the mitosRNAs are distributed in the mitochondrial and cytoplasmic compartments. N = normoxia; A = anoxia; Cytoplasm = non-mitochondrial cytoplasmic fraction.

**Figure 6:**
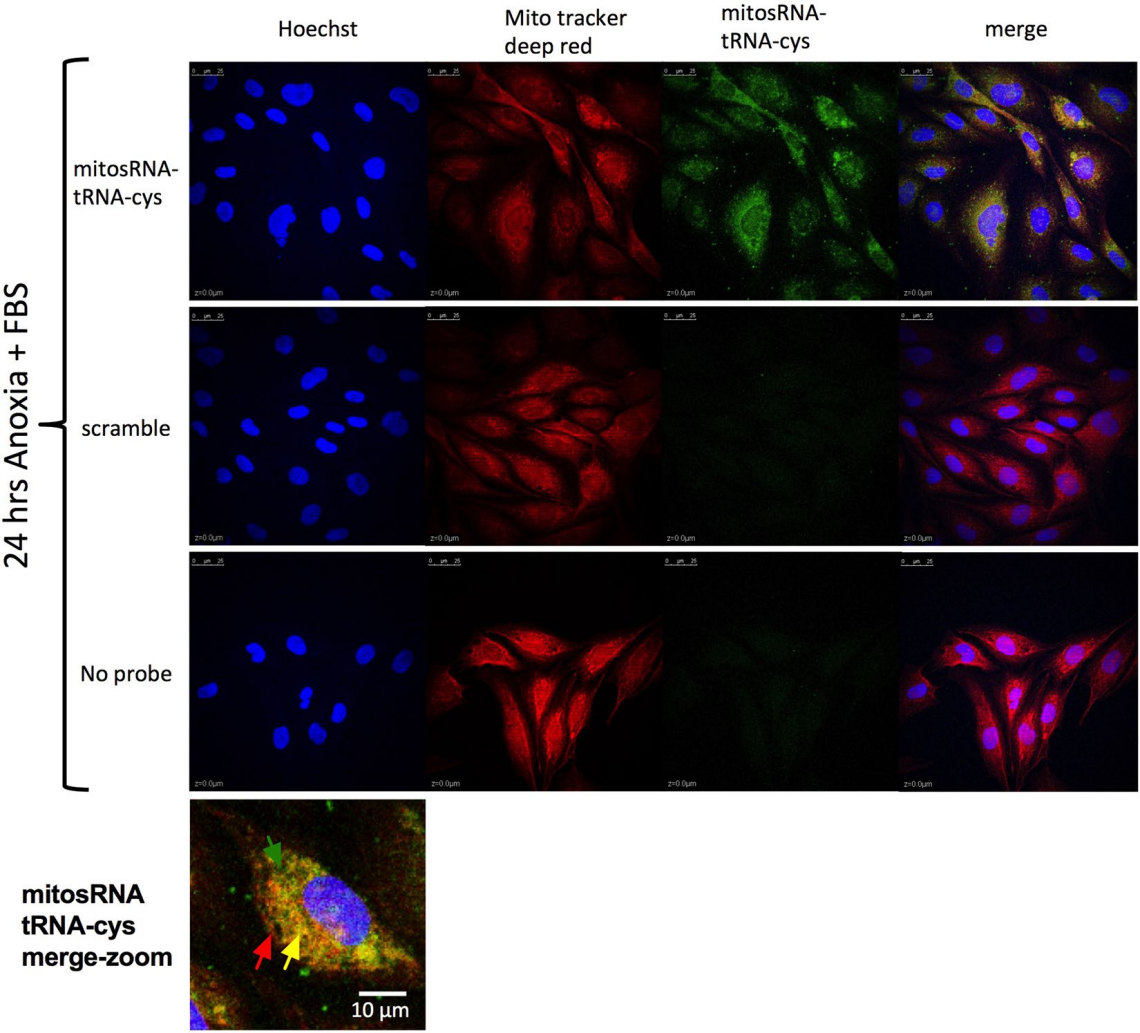
*In situ* hybridization of mitosRNA-tRNA-cys 3′ variants shows co-localization with mitochondria under anoxia. At 70–80% confluence, WS40NE cells were exposed to anoxia for 24 h and sampled for *in situ* hybridization. Anoxic cells were stained with Mitotracker Deep Red™ prior to *in situ* hybridization with mitosRNA-tRNA-cys probe, a scramble probe, or no probe. Cell nuclei were stained with Hoechst at the end of the procedure. Images show the three channels separately and merged for Hoechst, mitotracker, and the small ncRNA probe. Merged images reveal co-localization of mitochondria and mitosRNA-tRNA-cys. Colored arrows point to areas with mitochondrial signal (red), mitosRNA-tRNA-cys signal (green), or colocalization (yellow) on a merge imaged of a single cell.

Mature tRNA-cys and mitosRNA-tRNA-cys sequences were identified in subcellular fractions. Compared in parallel with whole cells, mitosRNA-tRNA-cys 3′ variants are particularly enriched in the mitochondrial fraction of WS40NE cells (Fig. 5a). (Note that direct comparison of the blots in 5a and 4b is not possible because different amounts of RNA were loaded and different amounts of probe were used. See figure legends for details). Additionally, mitosRNA-tRNA-cys 3′ variants appear in the cytoplasmic fraction (Fig. 5b). MitosRNA expression changes within subcellular compartments cannot be accurately assessed from the northern blots, as subcellular fractionation is not precise, and a single band can represent multiple mitosRNA variants, which, as previously mentioned, can differ in expression pattern. However, after 49 h anoxia, the dominant mitosRNA band in mitochondria and the cytoplasm (5b) is shifted up on the gel and does not align with normoxic mitosRNA bands, pointing to a change in expression within these subcellular compartments. MitosRNA-tRNA-cys was visualized throughout the cytoplasm in anoxic cells and co-localized (visible in yellow) with the mitochondria by *in situ* hybridization (Fig. 6). MitosRNA-tRNA-cys did not appear to localize to the nucleus, though the signal was strong around the nucleus.

### Inhibiting mitochondrial or nuclear transcription alters mitosRNA-tRNA-cys expression

**Figure 7:**
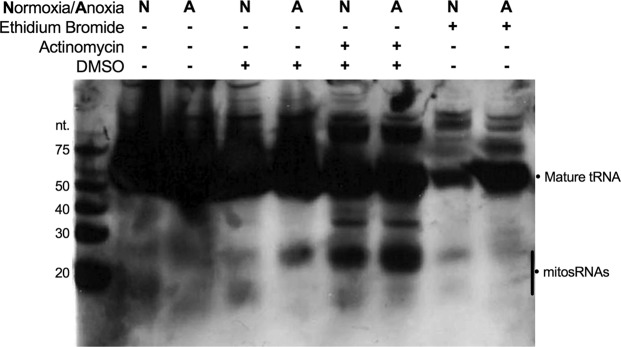
Northern blot for mitosRNA-tRNA-cys 3′ variants in cells treated with ethidum bromide or actinomycin to inhibit mitochondrial and nuclear transcription, respectively. Cells were pre-treated with ethidium bromide (0.4 µg/ml) to inhibit mitochondrial transcription or with actinomycin (10 µM) to inhibit nuclear transcription for 3 h prior to 24 h of anoxia or normoxia. Actinomycin-treated cells also received 0.1% of DMSO and thus cells treated with 0.1% DMSO were sampled as a control. “N” indicates normoxia and “A” indicates 24 h of anoxia. 20 µg RNA was loaded for each sample and the blot was hybridized with 2 nM mitosRNA-tRNA-cys probe.

MitosRNA-tRNA-cys were not clearly induced in response to anoxia in cells treated with ethidium bromide, an inhibitor of mitochondrial transcription (Fig. 7), compared to the clear induction observed in embryos and whole cells (Figs. 3d, 4b and Fig. 7 lanes 4 & 5; lanes 2 & 3 in Fig. 7 have high background and are not particularly informative). Inhibition of nuclear transcription with actinomycin treatment appears to increase expression of mitosRNA-tRNA-cys 3′-variants (Fig. 7), indicating that these small ncRNAs are not likely transcribed by the nuclear genome, and may even be induced by stresses other than anoxia.

### mitosRNAs localize to the trunk and head of the embryos

**Figure 8:**
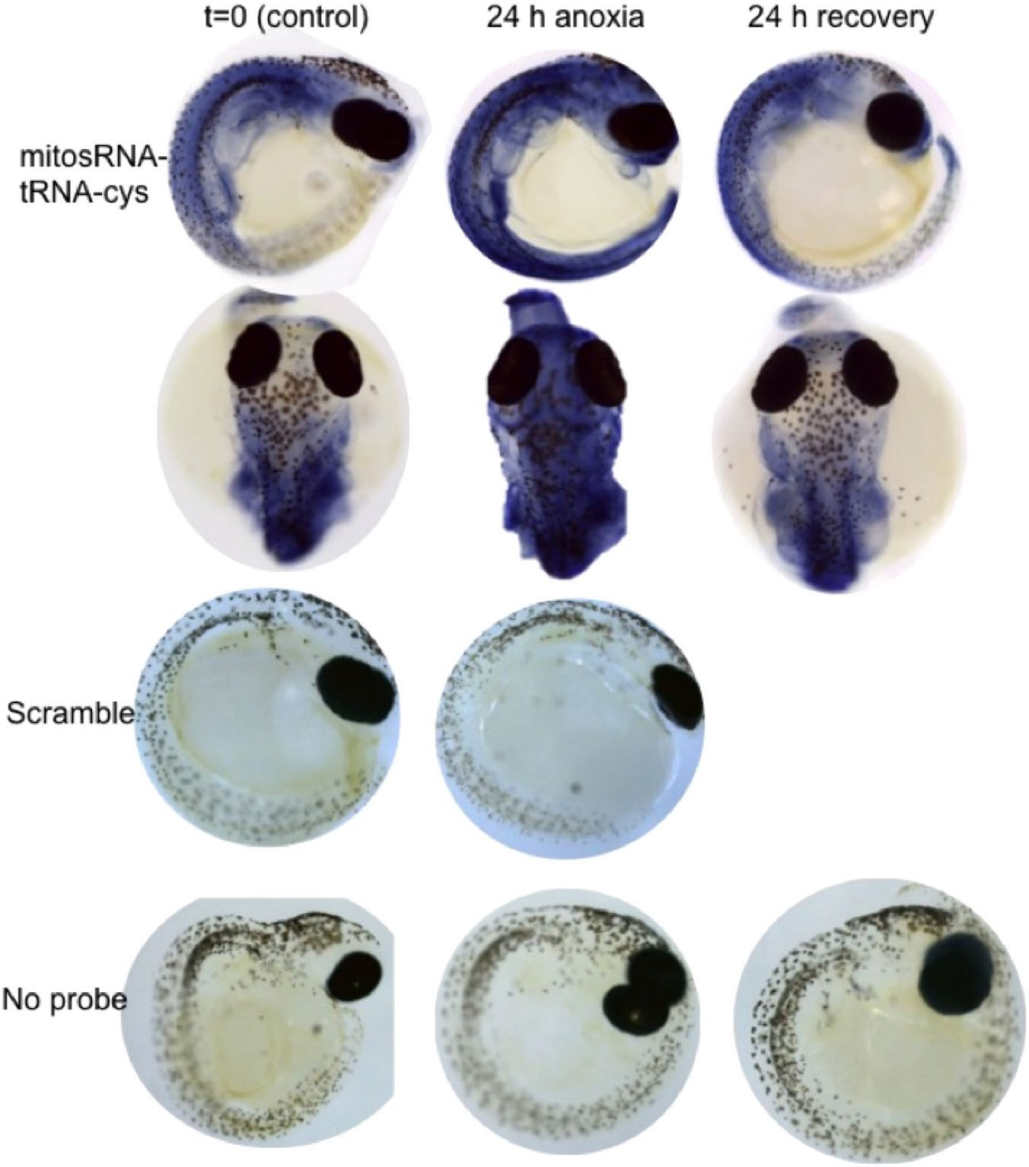
Whole mount *in situ* hybridization reveals mitosRNA-tRNA-cys 3′ variants throughout WS40 embryos. Embryos were sampled during normoxia, after 24 h anoxia, and 24 h aerobic recovery from anoxia. MitosRNA-tRNA-cys is expression increases across the entire embryonic axis during anoxia. As expected, embryos treated with scramble sequences or incubated without an RNA probe did not develop staining.

Highly differentially expressed mitosRNA-tRNA-cys was found throughout the body of WS 40 embryos (Fig. 8). This expression pattern is likely sequence-specific given that a scramble probe and no probe controls show very little staining. Under normoxia, expression was particularly strong around the developing gut, including the liver and kidney, and in the heart. In response to anoxia, expression of mitosRNA-tRNA-cys increased throughout the animal, especially in the brain. Expression patterns returned to pre-anoxic patterns after 24 h of recovery from anoxia.

## Discussion

Anoxia-responsive mitosRNAs in *A*. *limnaeus* are the first example of stress-responsive mitosRNAs. Expression of these sequences is positively correlated with anoxia tolerance and negatively correlated with metabolic rate of metabolically active embryos. They are particularly abundant during normoxia and differentially expressed in response to anoxia in WS 36 embryos, the most anoxia tolerant stage of actively developing embryos. A high proportion (~38%) of differentially expressed mitosRNAs align with mitochondrial tRNAs, and are highly differentially expressed in response to anoxia and recovery in WS 36 and WS 40 embryos, as well as the anoxia tolerant WS40NE cells derived from *A*. *limnaeus* WS 40 embryos. Further, a small subset of these sequences is constitutively expressed in diapause 2 embryos that are also highly induced during recovery from anoxia in WS 36 embryos. This pattern is indicative of a role in mediating recovery from anoxia. Diapause 2 embryos are already dormant when exposed to anoxia, thus their ability to respond with a large *de novo* response is very likely limited, and thus constitutive expression may be necessary^20^. In contrast, WS 36 embryos are actively developing, and presumably must actively respond to anoxic stress and aerobic recovery. Thus, the overall and specific expression of these sequences is associated with anoxia tolerance in a manner consistent with the unique physiology of the two most anoxia tolerant embryonic stages. This pattern of expression suggests an adaptive response to anoxic stress, as opposed to the most likely alternative explanations of disruption or dysregulation at the mitochondrial or RNA-level.

The changes observed in mitosRNA expression in *A*. *limnaeus* embryos in response to anoxia are not likely the result of changes in mitochondrial homeostasis, as previous work indicates that *A*. *limnaeus* embryos maintain mitochondrial homeostasis when faced with anoxia and subsequent recovery^21^. Mitochondrial DNA content (a proxy for the number of mitochondria) does not change in WS 36, WS 40, or WS 42 embryos in response to anoxic treatment followed by aerobic recovery. Citrate Synthase activity (a proxy for mitochondrial activity) also does not change significantly during anoxia in these same stages of embryos exposed to the same conditions. Further, mitochondrial tRNA transcript levels for tRNA-Cys (and all but 2 tRNA leucine genes) do not change in abundance during anoxia in diapause 2 embryos of *A*. *limnaeus*^22^. These data provide evidence for the maintenance of mitochondrial homeostasis in *A*. *limnaeus* during anoxia, making changes in mitochondrial number, condition, or global changes in gene expression unlikely explanations for the differential expression of mitosRNA sequences in response to anoxia. Additionally, the induction of different mitosRNAs displaying different expression patterns during anoxia and recovery (some starting abundant, while others scarce) further supports mitosRNA expression patterns as regulated processes, not artifacts from changes at the organelle level.

Given the overall stabilization of mitochondrial function during anoxia, it is unlikely that differential expression of mitosRNAs is due to global changes in the regulation of RNA degradation in mitochondria. If the tRNA-derived mitosRNAs described in this paper were the result of global tRNA degradation, we would expect to see an accumulation of fragments derived from all of the mature tRNAs in roughly equal proportions, which is not the case. Rather, particular tRNAs generate abundant and anoxia-responsive mitosRNAs. Additionally, RNA degradation pathways in vertebrate mitochondria remain largely undescribed. However, it appears that the major route for mammalian mitochondrial RNA degradation is via the 3′–5′exonuclease activity of polynucleotide phosphorylase (PNPase) that is part of the mitochondrial RNA degradosome^23^. Inhibition of PNPase activity in human mitochondria leads to the accumulation of RNA decay intermediates, many of which are antisense or “mirror” sequences to protein coding mt-mRNAs^24,25^. It is possible that anoxia blocks or reduces RNA degradation in *A*. *limnaeus* mitochondria based on observations in anoxic embryos of *Artemia franciscana* where COI mRNA is stabilized^26^. Indeed, the fact that only 208 of over 88,000 sequences are differentially expressed suggests a global stabilization of mitosRNA expression in response to anoxia and recovery from anoxia. Further, we do not observe accumulation of sequences from the light strand, the primary source of antisense or “mirror” sequences in mammalian systems, during anoxia in *A*. *limnaeus*. This suggests that anoxia-responsive mitosRNAs are generated by a process distinct from typical RNA degradation pathways. Additionally, if anoxia simply blocks RNA degradation in mitochondria from *A*. *limnaeus*, then we would expect to see the accumulation of these mitosRNAs in all 4 developmental stages examined. Further, arrest of general RNA degradation would likely lead to the accumulation of higher levels of degradation intermediates in the late-stage embryos that have significantly higher metabolic rates and rely heavily on aerobic metabolism^17^. Thus, the observed patterns of mitosRNA expression in response to anoxia are much more consistent with regulated production than with dysregulation of RNA degradation. Finally, arrest or incomplete arrest of RNA degradation should lead to accumulation of 5′ ends of mtRNA transcripts, given that the major route for RNA degradation is via a 3′-5′exonuclease. In contrast to this prediction, we observe many mitosRNAs that align with the 3′ end of the mature transcripts, which thus must be produced via an alternative mechanism. Unfortunately, little is known about RNA degradation in vertebrate mitochondria, and as of this point a mitochondrial 5′-3′exonuclease has not been specifically identified. Thus, identification of degradation pathways that could produce the full range of mitosRNAs in *A*. *limnaeus* remain to be identified.

Given the interest in mitosRNAs generated from the sequence expression data, and the unlikelihood of them being products of random degradation, we wanted to look for evidence for where they may be produced and active. Our results support the generation of mitosRNA-tRNA-cys variants from mitochondrial transcripts, but the details of biosynthesis remain unclear. The presence, and even increase in expression, of mitosRNAs in cells treated with an inhibitor of nuclear transcription provides strong evidence that these mitosRNAs are not transcribed in the nucleus, but rather the mitochondria. Furthermore, their expression may even be induced by the stress of inhibition of nuclear transcription suggesting that mitosRNAs may be part of a general stress response. Inhibition of mitochondrial transcription resulted in detection of less tRNA-cys derived RNAs all together (i.e. less total RNA on the blot), suggesting that the depletion of mitosRNAs may be due to a decline in overall transcription. The RNAs detected should represent RNA present and stabilized in the cells prior to exposure to ethidium bromide. This lens suggests that few mitosRNAs are present upon entry into anoxia when mitochondrial transcription is inhibited, and that the cells were not able to generate mitosRNAs under these conditions, as anoxia does not strongly induce mitosRNA expression in EtBr treated cells. Given the strong presence of the mature tRNA-cys in anoxic cells treated with mitochondrial transcription inhibitors, one would still expect the appearance of mitosRNAs if they were generated by cleavage of the mature tRNA. However, it is possible that ethidium bromide negatively affects tRNA stability as has been reported in other systems^23^, potentially altering the ability for tRNA cleavage to take place, and thus also causing the reduction of mitosRNAs. Another explanation (see above) is that the mitosRNAs are being preferentially degraded under normoxia, but not during anoxia. Without further experimentation, the mechanism of mitosRNA generation remains unclear.

Northern blot analysis and *in situ* hybridization both support localization of mitosRNA-tRNA-cys sequences to the mitochondrial and cytoplasmic compartments of the cell. Though cellular fractionation is imperfect and may result in bursting of some mitochondria, mitosRNA-tRNA-cys were clearly abundant in the cytoplasmic fraction, as the signal is too strong to be explained by burst mitochondria. In cells exposed to anoxia, the *in situ* hybridization pattern consists of overlap between the mitochondrial signal and the mitosRNA-tRNA-cys signal in much of the cell, particularly near the nucleus. However, some areas also show signal for mitosRNA-tRNA-cys and not for mitochondria, suggesting that these mitosRNAs may be exported from the mitochondria into the cytoplasm. Export of mature tRNAs or mitosRNAs from the mitochondria is plausible. For example, mitochondrial tRNA-methionine is exported to the cytoplasm and then associates with Argonaut, a protein involved in gene silencing^27^. Whether mitosRNAs are exported from the mitochondria or mature tRNAs are exported and then cleaved, the presence of mt-tRNA fragments in the cytoplasm suggests that these sequences may influence gene expression and physiology of the nuclear genome. Since the mitochondria are the major site of oxygen consumption in the cell, and mitochondria can sense limited oxygen and trigger signaling to the nuclear genome^28,29^, we hypothesize that mitosRNAs could be a component of this signaling. The dogma has been that the nuclear genome controls the mitochondrial genome, however, evidence of retrograde communication (from the mitochondria to the nucleus) is mounting^30,31^. Additionally, in a recent description of smithRNA (small mitochondrial highly-transcribed RNAs) the researchers hypothesize that these small ncRNAs may be exported from the mitochondria and act on nuclear gene expression, and discuss the idea that this could be a new form of retrograde communication^32^. Our data support this possibility in *A*. *limnaeus* cells, and add impetus for further experimentation to test this hypothesis.

The strong perinuclear co-localization signal of mitosRNA-tRNA-cys with mitochondria indicates that these mitosRNAs may function within mitochondria near the nucleus. A recent study found that perinuclear localization of mitochondria in response to hypoxia^33^ generates an ROS-rich environment in the nucleus, which leads to alterations in gene expression. Based on the presented image, it appears possible that mitochondria cluster near the nucleus in anoxic cells, yet further analysis is necessary to support this conclusion and investigate the perinuclear clustering of mitochondria under normoxic conditions. While more experiments are necessary, the presented data suggest there may be a relationship between perinuclear clustering, a potential increase in ROS, and mitosRNA co-localization with perinuclear mitochondria. Additionally, ROS patterns have yet to be studied in *A*. *limnaeus* embryos and cells, though a recent publication on *A*. *limnaeus* whole embryo antioxidant capacity reveals changes in antioxidant capacity associated with development and anoxia tolerance^34^, further implicating the need for a better understanding of ROS to develop a model of *A*. *limnaeus* cell biology in response to anoxia.

Nuclear mitochondrial pseudogenes (NUMTS) are an important consideration when describing new RNA species aligning to the mitochondrial genome. While it is *possible* that the mitosRNAs of interest discussed in this paper are actually pseudogenes encoded in the nuclear genome, based on resent publications from other groups, as well as evidence from our data, we find this unlikely. First, the mitosRNA-tRNA-cys was identified in the mitochondrial compartment in *in situ* hybridization and northern blots. Second, *in situ* hybridization does not support nuclear localization of these sequences. Third, inhibition of nuclear transcription does not cause a decrease but rather an increase in their expression. Additionally, our results are consistent with the findings of others. A recent in-depth analysis of mitosRNAs generated multiple lines of evidence supporting mitochondrial transcription of the sequences in question^35^. Additionally, analysis of mito-depleted human cells revealed a dramatic reduction in expression of mitosRNAs: only a few were expressed at low levels^11^, suggesting that most human mitosRNAs are not NUMTS. Thus, multiple lines of evidence support a mitochondrial origin for the mitosRNAs described in this paper.

While almost nothing is known about mitochondrial tRNA fragments, a great deal is known about nuclear tRNA-derived fragments (tRFs) and thus their biology may lend insight into potential routes for mt-tRNA fragment production, and their possible biological roles. Multiple mechanisms of nuclear tRF generation have been reported. MicroRNA biogenesis components, enzymes DICER and Argonaut, were identified in mitochondria of rat hippocampus^36^, though these same proteins are absent from mitochondria isolated from humans cells^11^, indicating that mitochondria from different species differ in which, if any, miRNA machinery components they contain. In mammals, the enzyme angiogenin cleaves mature tRNAs in response to stress, resulting in tRFs^37,38^. In other species, tRFs are generated by specific RNases such as by the miRNA-generating enzymes, drosha and DICER^39^. Ro *et al*.^11^ proposed that the production of mitosRNAs is likely due to activity of an unidentified mitochondrial ribonuclease. It is possible that a yet unidentified ribonuclease is also involved in mitosRNA generation in the annual killifish.

Nuclear tRFs respond to stress (increasing in expression) in a wide range of taxa, from yeast to plants and human cells^40–42^. Numerous stressors, including hypoxia and heat, as well as viral infection, induce the expression of nuclear tRFs^43^. It is possible that the stress-responsive mitosRNAs derived from tRNAs identified here may have a function similar to that of stress-responsive nuclear tRFs. In breast cancer cells, tRFs suppress the advancement of the cancer by destabilizing the expression of many pro-oncogenes^44^. Nuclear-derived tRFs also inhibit angiogenesis in response to ischemia^45^, and modulate cell proliferation and DNA damage^39^. In *Arabidopsis thaliana*, tRFs target transposable elements and help stabilize the genome under stress^46^, however this is not a suitable functional explanation for the mitosRNAs described here since they do not localize to the nucleus. Multiple examples document tRFs inhibiting protein synthesis. In human cells, tRNA fragments resulting from angiogenin cleavage of tRNAs inhibit translation initiation by interfering with the eIF4F complex^47^. In the halophilic archaeon *Haloferax volcanii* tRFs inhibit translation by binding to the ribosome^48^. Some 5′ tRFs can inhibit translation of reporter genes that don’t contain complementary sequence, indicating they are operating in a manner distinct from miRNAs^49^. While tRFs have diverse roles, it is interesting that inhibition of protein synthesis surfaces multiple times in the literature.

A role for mitosRNAs in protein synthesis inhibition fits with the biology of *A*. *limnaeus*. Given the localization data, it is possible that mitosRNAs inhibit mitochondrial protein synthesis, cytoplasmic protein synthesis, or both. Entry into metabolic depression is central to the ability of *A*. *limnaeus* to tolerate anoxia, and shutting down protein synthesis, both nuclear and mitochondrial, is a common component of metabolic depression^3,50–52^. It appears that mitosRNAs are associated with this entry into metabolic depression, especially since mitosRNAs are not differentially expressed in D2 embryos that are already metabolically depressed, but are very abundant and highly differentially expressed in WS 36 embryos, which have the same level of anoxia tolerance but are metabolically active prior to exposure to anoxia. Additionally, D2 embryos already have suppressed protein synthesis^51^, so if mitosRNAs are playing a role in suppression of protein synthesis their absence from D2 embryos follows logically. Given the expression of mitosRNAs in the context of *A*. *limnaeus* biology, and the preponderance of stress-responsive tRFs inhibiting protein synthesis in the literature, we hypothesize that anoxia-responsive mitosRNAs are involved in inhibition of protein synthesis in *A*. *limnaeus* embryos. Further experimentation will be necessary to test this hypothesis.

The mitosRNA signature in anoxia-tolerant *A*. *limnaeus* embryos strongly suggests mitosRNAs play an important role in the survival of anoxia in *A*. *limnaeus*. The diversity and abundance of mitosRNAs that are differentially expressed in response to anoxia and recovery, with a variety of expression patterns, lead us to hypothesize that these sequences could act in manifold mechanisms. We propose that in actively developing anoxia-tolerant *A*. *limnaeus* embryos, mitosRNAs are continuously generated by the mitochondrial genome, but that production increases during anoxia and recovery from anoxia. We hypothesize that mitosRNAs are found throughout the cells of the embryo and increase in expression globally in response to anoxia. Since they are highly differentially expressed in actively developing anoxia-tolerant embryos that require profound metabolic depression to survive anoxia, we hypothesize that these mitosRNAs play a role in coordinating metabolic depression, perhaps through suppression of protein synthesis. Based on the localization patterns, we hypothesize that these mitosRNAs affect mitochondrial and nuclear protein synthesis. Global inhibition of mitosRNA production, specific inhibition of individual mitosRNA activity, and over expression of individual mitosRNAs, coupled with anoxia tolerance assays, will be important in demonstrating the importance of mitosRNAs to the anoxia tolerance of *A*. *limnaeus* embryos and understanding their function. Immunoprecipitation experiments to investigate targets of these mitosRNAs will also be instrumental in understanding their utility. In conclusion, the data shown here reveal an exciting and potentially novel mechanism for supporting anoxia tolerance, and also suggest a new mechanism for retrograde communication between the mitochondria and the nucleus. Understanding the function of mitosRNAs has the potential to improve our understanding of anoxia tolerance and transform our knowledge of basic cellular biology. Given the exciting nature of the hypotheses surrounding mitosRNAs, building a strong foundation of research into their biology is critical. To this end, the work presented here includes an analysis of the expression patterns of mitosRNAs in anoxia-tolerant *A*. *limnaeus* cells and embryos, investigation into subcellular and organismal localization, and a preliminary look at the mechanisms of generation of mitosRNAs. We describe unique mitosRNA-cys expression patterns in the context of anoxia tolerance in the annual killifish and provide evidence for their enrichment in the brain and subcellular presence in the mitochondria and cytoplasm. Additionally, we provide evidence against nuclear transcription and in favor of mitochondrial transcription of the fragments in question. These data, taken together with literature of mitosRNAs and nuclear tRNA-derived fragments, suggest a functional role for mitosRNAs in mediating stress tolerance in embryos of *A*. *limnaeus*, but future studies will be needed to evaluate the possible mechanistic roles discussed in this paper.

## Methods

### Embryo maintenance and staging

Embryos of *Austrofundulus limnaeus* were collected from spawning adults and maintained using standard husbandry protocols^53^ and all procedures involving animals were conducted in accordance with Portland State University (PSU) Institutional Animal Care and Use Committee (IACUC protocol # 33). All work was performed according to relevant guidelines for the safe and ethical conduct of biological research involving recombinant or synthetic DNA/RNA as approved by the PSU Institutional Biosafety Committee (IBC protocol #1052). As previously described, embryos were staged and D2 embryos were exposed to 48 h of continuous light at 30 °C to break diapause, in order to obtain synchronous batches of post-D2 stage embryos^7^. Post-D2 embryos were staged according to morphology^16^. Morphological staging was based on annual killifish developmental stages described by J.P. Wourms^16,18^. This staging is useful since annual killifish embryo development can vary such that a given morphological stage is not always reached after the same number of days. Wourms’ stage (WS) 36 embryos were collected at 4 days post diapause 2 (dpd) and WS 40 embryos were collected at 12 dpd for whole embryo *in situ* hybridization and northern blots of small ncRNAs of interest, since these are the two most anoxia-tolerant metabolically active stages with the most robust mitosRNA response to anoxia.

### mitosRNA analysis

This paper represents the first in-depth focus on mitosRNA sequences in both whole embryos and in isolated cells from *A*. *limnaeus*. Sequence and expression data presented here represent a new analysis of small ncRNA sequence data sets from previously published work. Whole embryo mitosRNA sequence and expression data are from a large small ncRNA data set (NCBI Bioproject PRJNA272154) whose general characteristics have been previously described^7^. Expression data of mitosRNAs in the WS40NE cell line are also from a previously published data set (NCBI Bioproject PRJNA272154)^19^ and were re-analyzed for this publication. The data analysis pipeline used to generate expression data and differential expression analyses are reported in Riggs *et al*.^7^. To briefly summarize: (1) sequences were bioinformatically processed to remove adapters from low-quality reads from the dataset, (2) sequences were size selected to 15–27 nucleotides long, (3) sequences were retained that display 100% alignment to the *A*. *limnaeus* genome, (3) low abundance sequences were filtered out, (4) and finally libraries were normalized to account for sequencing depth differences (to make all RNA expression data comparable across treatments). Differentially expressed sequences (padj < 0.01, |log_2_ Fold change| > 2 and mean counts > 25) were identified using DESeq2^54^. Sequences were annotated by blast hits against databases of known RNAs (miRBase and RFAM). Sequence clustering (k-means using the Euclidean distance similarity metric) was based on expression patterns using Cluster 3.0^55^. Heatmaps, as well as other graphs, were generated using GraphPad Prism 7.0c. MitosRNA sequence data analysis generated a particular interest in mitosRNAs derived from tRNA-cysteine, as those variants were particularly abundant and differentially expressed (compared to other mitosRNAs), both in whole embryos and the WS40NE cell line.

### *In Situ* hybridization for mitosRNA-tRNA-cys

*LNA probes*. Linked Nucleic Acid (LNA) backbone probes for small ncRNAs of interest were ordered from Exiqon (Denmark, Germany). All probes were labeled with digoxigenin (DIG) at the 5′ and 3′ ends. The scramble was a standard probe for a sequence of nucleotides absent from the *A*. *limnaeus* genome, to serve as a control (5′-DIG-GTGTAACACGTCTATACGCCCA-DIG-3′; product no. 99004–15). The mitosRNA-tRNA-cys probe was custom designed to target a highly conserved region of the most differentially expressed mitosRNAs derived from tRNA-cys. These variants align to the 3′ end of the mature tRNA-cys. The mitosRNA-tRNA-cys probe (5′-DIG-AGTCCCGGCTGGCGAAT-DIG-3′) targets a conserved 17-nucleotide core shared by each 3′ variant. These LNA probes were used for whole embryo and cell *in situ* hybridizations and northern blots.

#### Embryo sampling for whole mount in situ hybridizations

Embryos were exposed to anoxia using a Bactron III anaerobic chamber (Sheldon Manufacturing, Cornelius, OR), followed by aerobic recovery as previously described^7^. Embryos were sampled under aerobic conditions (normoxia), and then after 4 and 24 h of anoxia, and 2 and 24 h of aerobic recovery from 24 h anoxia. These sampling times match the small ncRNA sequencing treatment time points^7^. Embryos were fixed overnight in 4% paraformaldehyde (PFA) in phosphate buffered saline (PBS, 2.7 mM KCl, 1.5 mM KH_2_PO_4_, 136.9 mM NaCl, 8.9 mM Na_2_HPO_4_, pH 7.4), rocking at 4 °C. For anoxia-exposed samples, samples were transferred to tubes with anoxic 4% PFA/PBS and sealed before removal from the anoxic chamber. In the Bactron anaerobic chamber, anoxic 4% PFA was prepared by diluting 32% PFA stored under a nitrogen atmosphere (Electron Microscopy Sciences Hatfield, PA) into PBS that was bubbled with N_2_ gas for 30 min prior to the addition of PFA. Following overnight fixation, embryos were dechorionated using fine forceps and were re-fixed in 4% PFA/PBS overnight at 4 °C. Embryos were rinsed of PFA and dehydrated in a methanol/PBS-T (PBS + 0.01% tween 20) dehydration series rinsing 5 min each at room temperature (RT) in 25% MeOH/75% PBS-T, 50% MeOH/50% PBS-T, 75% MeOH/72% PBS-T, and 100% MeOH, and stored at −20 °C until use for *in situ* hybridization.

#### Whole mount *in situ* hybridization

Whole mount small ncRNA *in situ* hybridization methods were adapted from a Bakkers lab protocol^56^. Stored embryos were rehydrated in a methanol/PBS-T (0.01% tween-20) series, rinsing 5 min each at RT in 75% MeOH/25% PBS-T, 50% MeOH/50% PBS-T, 25% MeOH/75% PBS-T, and 100% PBS-T. Rehydrated embryos were then permeabilized by incubating in proteinase K (10 µg/ml) for 10 min at RT. Permeabilized embryos were re-fixed in 4% PFA/PBS for 20 min at RT. Following fixation, embryos were rinsed in PBS-T (5 × 10 min). Embryos were prehybridized in Hyb + solution consisting of 50% deionized formamide (VWR, Radnor, PA), 5x sodium chloride/sodium citrate (SSC from G-Bioscience, St. Louis, M. O.), 0.1% tween-20 (Bio-Rad, Hercules, CA), 9 mM citric acid (VWR, Radnor, PA), 50 µg/ml heparin (Sigma, St. Louis, MO), 0.5 mg/ml tRNA from torula yeast (Sigma, St. Louis, MO). Embryos were prehybridized for at least 2 h at hybridization temperature (30 °C below the RNA melting point of the probe of interest). Fresh hybridization solution with probes for small ncRNAs were added to the embryos at a final probe concentration of 50 nM. Embryos were hybridized overnight followed by washes in Hyb– solution consisting of 50% deionized formamide, 5x SSC, 0.1% tween-20, and 9 mM citric acid at hybridization temperature for 10 min. Stringency washes (at hybridization temperature) to transition the buffer from Hyb– to 2X SSC were carried out for 10 min each as follows: 75% Hyb–/2x SSC-T, 50% Hyb–/2x SSC-T, 25% Hyb–/2x SSC-T, 2x SSC-T. The embryos were then washed twice for 30 min each in 0.2x SSC-T at hybridization temperature. Embryos were then transferred to PBS-T through a series of 10 min washes at RT (75% 0.2X SSC-T/25% PBS-T; 50% 0.2X SSC-T/50% PBS-T; 25% 0.2X SSC-T/75% PBS-T; 100% PBS-T). Embryos were subsequently incubated in 0.1 M glycine (Sigma, St. Louis, MO), pH 2.2 + 0.01% tween-20 for 45 min to deactivate endogenous alkaline phosphatases. Embryos were blocked for 1 h at RT in blocking buffer (2 mg/ml BSA (Sigma, St. Louis, MO), 5% normal sheep serum (Jackson ImmunoResearch, West Grove, PA) in PBS, followed by incubation in anti-digoxigenin-AP antibody (1:2000 in blocking buffer, Roche, Basel, Switzerland) with rocking at 4 °C overnight. Excess antibody was washed from embryos (3 × 5 min in PBS-T; 6 × 10 min PBS-T) at RT. Embryos were equilibrated to staining buffer containing 0.1 M tris-HCl (Sigma, St. Louis, MO), 0.05 M MgCl_2_ (Sigma, St. Louis, MO), 0.1 M NaCl (Sigma, St. Louis, MO), 0.1% tween-20 (Bio-Rad, Hercules, CA), 2 mM levamisole (Thermo Fisher Scientific, Waltham, MA) with one 5 min wash followed by two 15 min washes. Embryos were then stained with 75 mg/ml Nitrotetrazolium Blue Chloride (NBT, Sigma, St. Louis, MO) and 50 mg/ml 5-bromo-4-chloro-3-indolyl-phosphate, 4-toluidine salt (BCIP, VWR, Radnor, PA) in staining buffer and monitored on a steromicroscope. Once distinct staining was apparent, embryos were rinsed in PBS-T (3 × 5 min). Care was taken to stain all treatments for equal times. Incubation time was determined by the time for the stain to develop in embryos treated with the mitosRNA-tRNA-cys probe. After staining, embryos were re-fixed overnight in 4% PFA/PBS at 4 °C. Fixed embryos were rinsed in PBS-T (3 × 5 min), and dehydrated in methanol (25% MeOH/75% PBS-T, 50% MeOH/50% PBS-T, 75% MeOH/25% PBS-T, and 100% MeOH) at RT. After methanol dehydration, embryos were transferred to Murray’s clearing solution (2:1 benzyl alcohol:benzyl benzoate, Sigma, St. Louis, MO) and stored at 4 °C. Cleared embryos were imaged with a Leica stereomicroscope.

#### *In situ* hybridization in WS40NE cells

The cell *in situ* hybridization techniques were based on a double cell labeling protocol for miRNA and mitochondria in human cells^57^ and from miRNA *in situ* detection in mouse tissue sections^58^. WS40NE cells were grown to confluence in L-15 medium supplemented with 20% FBS (Thermo Fisher Scientific, Waltham, MA), 5 mM glucose (Sigma, St. Louis, MO), and 100 U/ml penicillin/streptomycin (Thermo Fisher Scientific, Waltham, MA) at 30 °C^19^. Confluent cells were split and seeded onto 12 mm glass coverslips in 12-well CytoOne tissue culture plates (USA Scientific, Ocala, FL) at a 1:54 split ratio. After cells adhered to coverslips overnight under normoxic conditions they were exposed to anoxia. Cells were introduced to anoxia in a Bactron III anaerobic chamber (Sheldon Manufacturing, Cornelius, OR), which maintained an atmosphere of 95% nitrogen and 5% hydrogen at 30 °C. Original media was removed from each well and replaced with 600 µl anoxic L-15 media supplemented with 8.5% FBS and 5 mM glucose^19^. Cells were kept in anoxia at 30 °C for 24 h. After 23.5 h anoxia, cells were stained with 3 µM Mitotracker Deep Red (Molecular Probes, Eugene, OR). After 30 min of staining (24 h anoxia), wells were rinsed with anoxic PBS. Following the rinse, anoxic 4% PFA/PBS was added to each well. The well plate was sealed with parafilm, removed from the chamber, and the cells were allowed to fix at 4 °C overnight. After fixation, cells were rinsed with PBS (3 × 5 min), permeabilized in chilled 100% methanol (VWR, Radnor, PA) at −20 °C for 10 min, and rinsed in PBS (2 × 5 min). Permeabilized cells were equilibrated for 10 min at RT to 1-MIB buffer containing 1.06% 1-methylimidazole (Roche, Basel, Switzerland), 318 µM NaCl, pH = 8.0. Cells were then fixed in 160 µM 1-Ethyl-3-(3-dimethylaminopropyl)carbodiimide (EDC, Sigma, St. Louis, MO) in 1-MIB buffer. Fixed embryos were washed with 0.2% Glycine (Sigma, St. Louis, MO) in PBS for 5 min, followed by washes in PBS (2 × 5 min). Cells were prehybridized for 4 h at RT in hybridization solution consisting of 50% deionized formamide, 5X SSC, 5X Denhardt’s solution (VWR, Radnor, PA), 500 µg/ml salmon sperm DNA (Thermo Fisher Scientific, Waltham, MA), 250 µg/ml yeast tRNA (Sigma, St. Louis, MO), 0.05 g/ml dextran sulfate (Santa Cruz Biotechnology, Dallas, TX), and 0.02 g/ml Roche Blocking Reagent (Sigma, St. Louis, MO), based on Obernosterer *et al*.^58^. Hybridization was carried out overnight at 37 °C in hybridization buffer with the addition of 0.25% CHAPS (VWR, Radnor, PA), 0.1% tween-20 (Bio-Rad, Hercules, CA), and 50 nM LNA probes. Probes were denatured prior to hybridization by heating to 90 °C for 5 min before plunging into ice water. Following overnight hybridization, coverslips were rinsed at 42 °C in 5X SSC for 5 min, followed by a 1 h incubation in 0.2X SSC at 42 °C. Cells were then rinsed in PBS (3 × 5 min). Cells were blocked for 1 h at RT in 10% FBS in PBS, and then incubated overnight in anti-Digoxigenin-AP (Roche, Basel, Switzerland) diluted 1:2000 in block buffer. Signal was amplified using FastRed (Sigma, St. Louis, MO), according to the manufacturer’s instructions. Nuclei were stained with 1 µg/ml Hoechst (Molecular Probes, Eugene, OR) prior to mounting in SlowFade^TM^ Gold Antifade Mounting medium (Molecular Probes, Eugene, OR). Slides were imaged with a TCS SPE II confocal microscope (Leica, Wetzler, Germany).

### Northern blots

Northern blots were used to qualitatively assess the presence/absence of mitosRNAs under different conditions and in different subcellular compartments in annual killifish embryos and cells. It is important to note that the probe designed to target mitosRNA-tRNA-cys variants aligning to the 3′ end of mature tRNA-cys will bind to any of the mitosRNA-tRNA-cys variants containing the core sequence targeted by the probe. While enabling the visualization of a size range of RNAs containing the core sequence (including the mature tRNA), this approach also results in the probe binding to various unique mitosRNA sequences of similar size, resulting in bands comprised of multiple RNAs, making the northern blot particularly ill-suited for quantification. However, the northern blot is a useful tool for assessing expression pattern trends, and subcellular localization.

### Cell and whole embryo treatment and sampling for northern blots

WS40NE cells were maintained at 30 °C in complete cell culture medium (L-15 medium supplemented with 8.5% FBS, 5 mM glucose, and 100 U/ml penicillin/streptomycin) as previously described^19^. Cells were grown to confluence in 100 mm plates and exposed to treatment (anoxia or normoxia) for 24 or 49 hrs prior to sampling for RNA extraction. For RNA extraction, 7 ml TRIzol™ (Thermo Fisher Scientific, Waltham, MA) was added directly to each plate and RNA extraction was completed according to the manufacturer’s instructions. Embryos for northern blots were exposed to anoxia as for the whole mount *in situ* hybridization samples, but were flash frozen at the designated sampling point and stored at −80 °C prior to extracting RNA as previously described^7^.

Northern blotting of mitosRNAs was performed following protocols outlined in Kim *et al*.^59^, with slight modifications. RNA samples were diluted in gel loading buffer II (Thermo Fisher Scientific, Waltham, MA) 3–20 µg RNA per lane were loaded on and run on a 15% polyacrylamide, 7.5 M urea gel (National Diagnostics, Atlanta, GA) and transferred to a positively charged nylon membrane (Sigma, St. Louis, MO) by capillary action with 20X SSC (G-Biosciences, St. Louis, MO) and a TurboBlotter (GE Healthcare) transfer system. RNA was UV cross-linked to the membrane for 4 min at 120,000 µJ/cm^2^ using a UVP Hybrilinker. The nylon membrane was prehybridized in ULTRAhyb ultrasensitive hybridization buffer (Thermo Fisher Scientific, Waltham, MA) and incubated overnight at 37 °C in hybridization solution with 0.5–2 nM LNA probes (see *LNA probes* section for sequence and DIG labeling). Hybridization solution was rinsed from the membrane with 2 × 15 min low stringency washes (2x SSC, 0.1% SDS), 2 × 5 min high stringency washes (0.1x SSC, 0.1% SDS), and a 10 min wash in 1x SSC. All washes were performed at 37 °C. The membrane was blocked for 3 h at RT in blocking solution consisting of 0.1 M maleic acid, 0.15 M NaCl, 0.3% Tween-20, 2 mg/ml BSA, and 5% normal sheep serum (Jackson ImmunoResearch, West Grove, PA) prior to a 30 min incubation at RT in anti-digoxigenin-AP antibody (Roche, Indianapolis, Indiana) diluted 1:10,000 in blocking solution. The membrane was washed 4 × 15 min at RT in DIG washing solution from the DIG Wash and Block Kit (Sigma, St. Louis, MO). The membrane was equilibrated in 1x Detection Buffer from the DIG Wash and Block Kit (Sigma, St. Louis, MO). Signal was detected using Disodium 3-(4-methoxyspiro {1,2-dioxetane-3,2′-(5′-chloro)tricyclo [3.3.1.1^3,7^]decan}-4-yl)phenyl phosphate (CSPD, Sigma, St. Louis, MO), diluted 1:100 in Detection Buffer. Blots were incubated in CSPD solution for 15 min in the dark prior to imaging. Blots were exposed to film for 15 min and developed manually. It is important to note that amount of RNA loaded and probe concentration were varied among figures, depending on the optimal conditions for the given samples. For whole embryo RNA samples, 3 µg RNA was loaded and hybridized with 0.5 nM probe. Since the mitosRNA signal is strong in whole embryos, these concentrations were appropriate. For whole cell experiments it was necessary to load 20 µg RNA and hybridized with 2 nM probe in order to clearly detect the mitosRNA signal in WS40NE cells, which have a less robust mitosRNA signal compared to whole embryos. In subcellular fractionation experiments, 5 µg RNA was loaded and hybridized with 0.5 nM probe. Since some of the subcellular compartments are enriched in these mitosRNAs of interest, less RNA (compared to the whole cells) was needed to detect the bands.

### Inhibition of nuclear and mitochondrial transcription to assess mitosRNA biosynthesis

Transcription inhibition studies in conjunction with northern blots were conducted to assess the possible contributions of nuclear and mitochondrial transcription to generating mitosRNAs. WS40NE cells were grown to confluence in complete cell culture medium as previously described^19^. Upon reaching confluence, cells were treated with 0.4 µg/ml ethidium bromide (Sigma, St. Louis, MO) to inhibit mitochondrial transcription^60^ or with 10 µM actinomycin and 0.1% DMSO (Enzo Life Science, East Farmingdale, NY) to inhibit nuclear transcription per the manufacturer’s recommendations. Actinomycin effectively inhibits transcription of different RNA species, including tRNAs^61^. Control samples for actinomycin-treated cells were treated with 0.1% DMSO. Cells were pre-incubated in inhibitors for 3 h, after which the cells for the anoxic treatment were transferred into the anoxic chamber. Once in the chamber the original media was replaced with anoxic media (L-15 supplemented with 8.5% FBS, 100 u/mL pen-strep, and 5 mM glucose) with the appropriate concentrations of ethidum bromide, actinomycin, or DMSO to match pre-treatment/normoxic conditions. Cells were incubated under experimental conditions for 24 h prior to extraction of total RNA. Total RNA was extracted and purified by adding Trizol reagent directly to the well to lyse the cells and then following the manufacturer’s instructions for RNA purification.

#### Subcellular fractionation

24 plates of cells were grown to confluence in 100 mm CytoOne cell culture treated plates (USA Scientific, Ocala, FL) in L-15 medium supplemented with 8.5% FBS, 5 mM glucose, and 100 U/ml penicillin/streptomycin. 12 plates were transferred into the anaerobic chamber and media was replaced with anoxic L-15 medium supplemented with 8.5% FBS, 5 mM glucose, and 100 U/ml penicillin/streptomycin. The remaining 12 plates were kept in the aerobic incubator at 30 °C in original media. After 24 h, cells from all plates were harvested by mechanical scraping. 1.5 ml ice-cold PBS containing 2.7 mM EDTA was added to each 100 mm plate of cells and allowed to incubate for 5 min. In the case of anoxic cells the PBS solution was anoxic. Following a 5 min incubation, cells were scraped off the plate with a plastic policeman and collected into two 15 ml conical tubes. Cells were pelleted by centrifugation at 100 × *g* at 4 °C for 7 min to prepare for mitochondrial isolation according to Duerr and Podrabsky 2010^62^ who previously established the method in *A*. *limnaeus*. The supernatant was poured off and the pellet was resuspended in 2.5 ml (about 10X the pellet volume) of mitochondria isolation buffer (117 mM KCl, 20 mM TES, 5 mM EGTA, 2% fatty acid free BSA). Cell suspensions for the same treatment were combined in a Wheaton dounce homogenizer. The suspension was homogenized with a loose-fitting Teflon pestle for 15 strokes, followed by 15 strokes with a tight-fitting pestle. The solution was poured into a round bottom conical tube and subjected to centrifugation at 780 × *g* at 4 °C for 10 min to pellet whole cells and nuclei. The supernatant was transferred to a 15 ml conical tube and centrifuged for 10 min at 10,000 × *g* at 4 °C to pellet the mitochondria. The supernatant containing cytoplasmic components and organelles smaller than mitochondria was transferred to a 15 ml conical tube. Mitochondrial and whole cell/nuclei pellets were resuspended in 1 ml TRIzol™ (Thermo Fisher Scientific, Waltham, MA). TRIzol LS Reagent™ (Thermo Fisher Scientific, Waltham, MA) was added to the cytoplasmic fraction according to the manufacturer’s instructions. All samples were stored at −20 °C until RNA extraction according to the manufacturer’s instructions.

## Data Availability

The datasets generated during and/or analyzed during the current study are available in the National Center for Biotechnology Information (NCBI) repository, https://www.ncbi.nlm.nih.gov/bioproject/272154.
